# Quantum hash function based on controlled alternate lively quantum walks

**DOI:** 10.1038/s41598-023-33119-w

**Published:** 2023-04-11

**Authors:** Penglin Hou, Tao Shang, Yuanjing Zhang, Yao Tang, Jianwei Liu

**Affiliations:** School of Cyber Science and Technology, Beihang University, Beijing, 100083 China

**Keywords:** Computer science, Quantum information

## Abstract

Quantum hash function is an important area of interest in the field of quantum cryptography. Quantum hash function based on controlled alternate quantum walk is a mainstream branch of quantum hash functions by virtue of high efficiency and flexibility. In recent development of this kind of schemes, evolution operators determined by an input message depend on not only coin operators, but also direction-determine transforms, which usually are hard to extend. Moreover, the existing works ignore the fact that improper choice of initial parameters may cause some periodic quantum walks, and further collisions. In this paper, we propose a new quantum hash function scheme based on controlled alternate lively quantum walks with variable hash size and provide the selection criteria for coin operators. Specifically, each bit of an input message determines the magnitude of an additional long-range hop for the lively quantum walks. Statistical analysis results show excellent performance in the aspect of collision resistance, message sensitivity, diffusion and confusion property, and uniform distribution property. Our study demonstrates that a fixed coin operator, along with different shift operators, can effectively work on the design of a quantum hash function based on controlled alternate quantum walks, and shed new light on this field of quantum cryptography.

## Introduction

Hash function plays an important role in modern cryptography, which is considered a key component of almost all encryption schemes and many security applications, such as message authentication code, data integrity, data storage, and random number generation. In the classical context, hash function can map a message of arbitrary length to a fixed-size output. Generally, there are two types of hash functions in terms of security attributes. One is the collision-resistant hash function that has provable security reductions and the other is based on an iterated construction of compression functions. The former satisfies computational security but is too inefficient to be used in practice. Some provably secure examples can reduce to hard problems, such as the $\textit{integer factorization problem}$^1^, the $\textit{very smooth number nontrivial modular square root problem}$ (VSSR)^2^, and the $\textit{Knapsack problem}$^3^. The latter is easily designed and more efficient because the design of hash functions can be transformed into that of compression functions^4^. Meanwhile, cryptanalysis attacks also have gradually emerged in recent years, which poses a huge challenge to the security of such hash functions^5^.

With the development of quantum computing, more and more researchers have shown increasing interests in using quantum mechanisms to develop cryptographic algorithms with high security, especially the quantum hash function, which is one of the most important in cryptographic discussions^6–23^. There are two approaches of research on quantum hash functions. One is based on quantum one-way functions (QOWF)^6–11^, while the other one is based on quantum simulation, including discrete-time quantum walk (DQW)^13–21,23^, continuous-time quantum walk (CQW)^22^ and grouped coarse-grained boson sampling (GCGBS)^12^. The former has attempted to formally define the one-way and collision resistance property of quantum hash functions, which have been conclusively proved with a rigorous mathematical form. The latter constructs quantum hash functions by virtue of quantum mechanics, specifically, the chaotic characteristic of quantum walk or the irreversibility of GCGBS without formal security proof. Alternatively, one can evaluate such types of schemes through statistical analysis which can provide quantitative evidence. Moreover, QOWF-based quantum hash functions map a classical bit string to a quantum state whose lengths are positively related to the length of an input message. By contrast, DQW-based, CQW-based and GCGBS-based quantum hash functions generate a fixed-length classical hash value from a classical bit input. Of particular concern is QOWF-based schemes are only guaranteed to correctly distinguish the quantum hash states of two different input messages with a high probability by means of SWAP test, which means such schemes are non-deterministic with a one-side error. In addition, GCGBS-based quantum hash function could only be efficiently calculated through a linear optical network, but is awkward to perform on a classical computer due to high time complexity. In accordance with the above, the practical value of DQW-based and CQW-based quantum hash functions are higher compared to those based on QOWF and GCGBS, because the former has deterministic outputs for fixed inputs and can be simulated classically before the advent of commercial quantum computers.

As we know, the construction of quantum walks based quantum hash function relies on different quantum walks procedures with various unitary evolution operators which can be generally decomposed into coin and shift operators. Such schemes begin with an initial quantum state, then the evolution of the system proceeds by repeatedly using different unitary operators controlled by the input bit string, it will then obtain a classical hash value by post-processing algorithm, i.e., modular and truncation arithmetic. Li et al.^23^ explored, for the first time, a brand-new perspective on the design of a dedicated hash function based on quantum walks. They presented a quantum hash function whose underlying model is one-dimensional two-particle controlled interacting quantum walks (CIQW), and discussed the security and feasibility of the scheme. Over the past decade, more information has become available on the quantum hash functions based on quantum walks, particularly for the quantum hash functions based on controlled alternate quantum walks (CAQW)^13,15–17,19^, which are the improvement and extension of CIQW-based quantum hash functions^21,23^. Until now, almost all such schemes control the evolution of quantum walks by alternate coin operators, little attention has been paid to the role of shift or other operators besides coin operator. Recently, an extra operator called the direction-determine transform has been introduced in the work of Zhou et al.^15^, which can also be considered for a CAQW-based quantum hash function. In order to explore whether more operators are valid for designing a CAQW-based quantum hash function, we introduce lively quantum walks with variable liveliness parameters to construct a new CAQW-based quantum hash function.

The main contributions of this paper are described as follows:

1. Construction of a novel quantum hash function with variable hash size based on controlled alternate lively quantum walks with variable liveliness parameters, named QHFL. Also, the conditions for a periodic quantum walk are discussed, and used to design a secure CAQW-based quantum hash function.
2. Verification for feasibility and security of the proposed quantum hash function in both quantum and classical environments. Through theoretical analysis and statistical experiments, the results demonstrate the proposed scheme has an excellent performance and its scalability is better than that of an existing state-of-the-art CAQW-based quantum hash function^15^. Moreover, an efficient quantum circuit is given for implementation.

The rest of this paper has been organized in the following way: The “Preliminaries” introduces necessary preliminaries about the notion of hash function and quantum walk. The “Methods” is concerned with the scheme named QHFL. In the “Security analysis” and the “Statistical performance analysis”, the security analysis and statistical performance of QHFL are discussed, respectively. In the “Computing complexity”, both classical and quantum computing Complexity is offered. In the “Advantage”, the advantages of QHFL is are briefly discussed. Finally, the “Discussion” gives a summary of this work and provides directions for further research.

## Preliminaries

### Cryptographic hash function

A cryptographic hash function must satisfy three criteria: preimage resistance, second-preimage resistance and collision resistance.

- Preimage resistance
  A hash function is a one-way function. For any valid output, it is computationally infeasible to find the corresponding input. In particular, a function mapping $f:\left\{ 0,1 \right\} ^{*} \rightarrow \left\{ 0,1 \right\} ^{*}$ is a strong one-way function if it satisfies all of the following conditions:
  *Easy to calculate*: *f* can be computed in polynomial time.
  *Hard to invert*: A polynomial $p(\cdot )$ exists such that for any polynomial time adversary’s algorithm *A*, in the case of sufficiently large number $n\in {\mathbb {N}}$, has
  1 $$\begin{aligned} Prob[A(f\left( x\right) ,1^n)\in f^{-1}(f\left( x\right) )]<\frac{1}{p(n)} . \end{aligned}$$
  Given a message digest *y*, it is difficult to find its preimage *x* such that $H\left( x\right) =y$, instead, its inverse process is computable.
- Second-preimage resistance
  Finding any two inputs that have the same output is computationally infeasible, i.e., given an input *x*, find its second preimage $x'$ such that $H(x)=H(x')$.
- Collision resistance
  Finding two different inputs *x* and $x'$ such that their hash values are the same is computationally infeasible, i.e., $H(x)=H(x')$. In fact, collision resistance implies second-preimage resistance but does not guarantee preimage resistance.

### Lively quantum walk on an *N*-length cycle

Standard discrete-time quantum walk (DQW) is a quantum analogue of classical random walk, taking place in the product space ${\mathscr {H}}_p\bigotimes {\mathscr {H}}_c$ in which a particle (walker) is placed at a vertex in a cycle and allowed to jump along the edges to adjacent vertexes. Here a particle starts from its initial state $\left| x \right\rangle \otimes \left| c\right\rangle$, where the spatial degree of freedom $\{\left| x\right\rangle ,x\in {Z}_N \}$ spans the position space ${\mathscr {H}}_p$, and the two-dimensional coin state $\left\{ \left| c\right\rangle ,c\in \left\{ 0,1\right\} \right\}$ spans the coin space ${\mathscr {H}}_c$.In DQW on an *N*-length cycle, each evolution of a system proceeds by a unitary operator, which is defined as $U=S\left( I_N\otimes C\right)$, where *S* is the shift operator updating the position chirality states of the particle, *C* is the coin operator on the coin space ${\mathscr {H}}_c$ determining the direction at each walking step, and $I_N$ is the *N* order identity matrix of ${\mathscr {H}}_p$ space.The shift operator is defined as follows:

2 $$\begin{aligned} S=\sum _{x\in Z_N}{{\left| x+1 ({\rm{mod}} \ N)\right\rangle \langle x\vert \otimes \left| 0\right\rangle \langle 0 \vert +\left| x-1 ({\rm{mod}} \ N)\right\rangle \langle x\vert \otimes \left| 1\right\rangle \langle 1 \vert \ }}, \end{aligned}$$

then the evolution process of quantum walk can be defined in the situation by shift operator

3 $$\begin{aligned} S\left( \left| x\right\rangle \otimes \left| 0\right\rangle \right) =\left| x+1 ({\rm{mod}} \ N)\right\rangle \otimes \left| 0\right\rangle , S\left( \left| x\right\rangle \otimes \left| 1\right\rangle \right) =\left| x-1 ({\rm{mod}} \ N)\right\rangle \otimes \left| 0\right\rangle . \end{aligned}$$

Such operator implies that the particle moves from one vertex to the left or right corresponding to the coin quantum state $\left| 0\right\rangle$ or $\left| 1\right\rangle$, respectively.

One-dimensional three-state quantum walks(or lazy quantum walks), the generalized Hadamard walks(DQW) with three inner coin states, were first proposed by Konno et al.^24^. In recent years, Sadows et al.^25^ introduced a new family of quantum walks on cycles parametrized by their liveliness (named lively quantum walks). The introduced family contains lazy quantum walks, which can be considered as lively quantum walks with liveliness equal to zero.

Consistent with DQW, the process of a lively quantum walk on an *N*-length cycle occurs in Hilbert space ${\mathscr {H}}_p\otimes {\mathscr {H}}_c$, where ${\mathscr {H}}_p$ represents the position space, whose orthogonal basis is given by the label of each vertex on the *N*-length cycle, and defined as ${\mathscr {H}}_p=Span\left| x\right\rangle$, $x\in {Z}_N$. ${\mathscr {H}}_c$ represents the three-dimensional coin space, which is defined as ${\mathscr {H}}_c=Span\left| c\right\rangle , c\in \{\left| 0\right\rangle , \left| 1\right\rangle , \left| 2\right\rangle \}$. Then, a formulation for the lively quantum walk can be defined.

The shift operator *S* of the lively quantum walk acting on each vertex *x* is given by

4 $$\begin{aligned} \begin{aligned} S&=\sum _{x\in z_N}{ \left| x+1({\rm{mod}} \ N) \right\rangle \langle x\vert \otimes \left| 0 \right\rangle \langle 0\vert }\\&\quad +\left| x-1({\rm{mod}} \ N) \right\rangle \langle x\vert \otimes \left| 1 \right\rangle \langle 1\vert \\&\quad +\left| x+ \tau ({\rm{mod}} \ N) \right\rangle \langle x\vert \otimes \left| 2 \right\rangle \langle 2\vert , \end{aligned} \end{aligned}$$

with a liveliness parameter $\tau \le \left[ \frac{N}{2}\right] (\left[ \cdot \right]$ represents a ceiling function), which controls the particle to make an additional choice of hop. Thus, the entire evolution of the quantum system at each time step can be described by $U=S\left( I_N\otimes C\right) \in {\mathscr {H}}_p\ \bigotimes {\mathscr {H}}_c$, with a usually used coin operator called Grover operator *G*. $G=2\left| s_v\right\rangle \langle s_v\vert -I$, where $\left| s_v\right\rangle =\frac{1}{\sqrt{3}}(\left| 0 \right\rangle +\left| 1 \right\rangle +\left| 2 \right\rangle )$, and we can obtain $$G=\left[ \begin{matrix}-\frac{1}{3}&{}\frac{2}{3}&{}\frac{2}{3}\\ \frac{2}{3}&{}-\frac{1}{3}&{}\frac{2}{3}\\ \frac{2}{3}&{}\frac{2}{3}&{}-\frac{1}{3}\\ \end{matrix}\right]$$.

### Periodic quantum walk on a cycle

Under certain conditions, some coin operators may give rise to periodic quantum walks. Here, some necessary conditions for specific quantum walks with periodicity^26–30^ have been discussed. In particular, for the lazy quantum walk, Konno and Kajiwara^29^ studied the necessary condition that the coin operator to have a finite period. For a more general case, the periodicity phenomenon for lively quantum walks on cycles, generated by the combination of variable parameters of liveliness and generalized Grover coins, have been thoroughly studied. Sarkar and Mandal^30^ regarded the coin operator as a linear combination of orthogonal permutation matrices, extending the study of periodic lively quantum walk. Permutation matrices of this type are called Grover-type matrices, see A for details. According to Ref.^27^, the periodic quantum walk on a cycle can be defined as follows:

#### Periodic quantum walk on a cycle

Define a set as ${\mathscr {N}}=\{n \ge 1 \in {\mathbb {N}}: U^n={\mathbb {I}}\}$, where ${\mathbb {N}}$ is the set of nature numbers, *U* is the evolution operator corresponding to the quantum walk on a cycle, and ${\mathbb {I}}$ is an identity matrix. If ${\mathscr {N}}\ne \emptyset$, the period *T* of the quantum walk on a cycle is written as $min{{\mathscr {N}}}$. Otherwise, for the case of ${\mathscr {N}}=\emptyset$, the quantum walk on a cycle has a period defined as $\infty$ and is called an aperiodic quantum walk. In particular, the periodicity for quantum walk on a cycle means that the particle returns precisely to its quantum initial state after a finite number of steps.

## Methods

In this section, we introduce the controlled alternate lively quantum walk with variable liveliness on a cycle, then give the specific scheme for the quantum hash function based on it, named QHFL.

### Controlled alternate lively quantum walk on an *N*-length cycle

The controlled alternate lively quantum walk can be constructed by cleverly modifying lively quantum walk on an *N*-length cycle. Assume that the particle performs the lively quantum walk on an *N*-length cycle with liveliness parameter $\tau$. At the *t*-th time step, the quantum state amplitude of a particle at the position *x* is given by $$\left| \psi _{x,t}\right\rangle =\left[ \begin{matrix}a_{x,t}^1&{}a_{x,t}^2&{}a_{x,t}^3\\ \end{matrix}\right] ^\dag =\ \sum _{c}{a_{x,t}^c\left| c\right\rangle }\in {\mathscr {H}}_c$$, where $a_{x,t}^c$ represents the quantum state amplitude of the particle in the coin register, so the state of the whole system can be defined as

5 $$\begin{aligned} \left| \Psi \left( t\right) \right\rangle =\sum _{x\in z_N}{\left| x\right\rangle \otimes \left| \psi _{x,t}\right\rangle .} \end{aligned}$$

For the initial state $\left| \Psi \left( t=0\right) \right\rangle$ with a unitary operator *U* to update the quantum state at each step, the time evolution of the quantum state is defined as $\left| \Psi \left( t\right) \right\rangle =U^t\left| \Psi \left( t=0\right) \right\rangle$. Thus, the transformation of amplitude for the particle at the position *x* can be obtained as follows

6 $$\begin{aligned} \left| \psi _{x,t+1}\right\rangle =C_1\left| \psi _{x+1,t}\right\rangle +C_2\left| \psi _{x-1,t}\right\rangle +C_3\left| \psi _{x+\tau ,t}\right\rangle , \end{aligned}$$

where $C_i=\sum _{k=1}^{3}{{\tilde{C}}_{i,k}\left| i\right\rangle \left\langle k\right| },i\in \{1,2,3\}, {\tilde{C}}_{i,k}$ is the element in the *i*-th row and *k*-th column of matrix *C*. Now we can introduce the controlled alternate lively quantum walk with liveliness on a cycle.

Specifically, given an input message *msg*, each bit $\{{m}_1,m_2,\ldots m_t\}\in {\{0,1\}}^t$ of the message is a string of binary numbers, so the quantum walk evolution operator controlled by *t* bits message is $U_{msg}=U_{m_1}U_{m_2}U_{m_3}\cdots U_{m_t}$, where $U_{m_j}(1\le j\le t)$ denotes the evolution operator of *j*-th step which can be designed with variable liveliness parameters given by Eq. (4). Accordingly, the final quantum state can be expressed as $\left| \Psi \right\rangle _{final}=U_{msg}\left| \Psi \left( t=0\right) \right\rangle$. Then, after *t* steps, the probability of finding the particle being in the position *x* is given by

7 $$\begin{aligned} p_x=\left\langle \psi _{x,t}\vert \psi _{x,t}\right\rangle =\sum _{c=1}^{3}\left| a_{x,t}^c\right| ^2, \end{aligned}$$

satisfying the condition $\sum _{c=1}^{3}\sum _{x\in \ z_N}\left| a_{x,t}^c\right| ^2=1$, where $a_{x,t}^c$ can be obtained by means of measuring the basis state $\left| x,c\right\rangle$,

8 $$\begin{aligned} \left| \left\langle x,c\left| \left( U^t \right| \Psi \left( t=0\right) \right\rangle \right) \right| ^2. \end{aligned}$$

### Quantum hash function based on controlled alternate lively quantum walks

For an initial state $\left| \Psi \left( t=0\right) \right\rangle =\left| x\right\rangle \otimes \left| c\right\rangle$, where $\left| c\right\rangle =a_1\left| 0\right\rangle +a_2\left| 1\right\rangle +a_3\left| 2\right\rangle$, taking message *msg* as an input, the specific steps of QHFL can be described as follows:

1. Select a set of initial parameters $\left( N,l,s,C,a_1,a_2,a_3\right)$, where *N* is an integer, representing the number of vertexes in a cycle, *l* represents the amplification factor of the probability with the constraint ${10}^l\gg 2^s$, *C* is a coin operator selected from a set that meets the security requirement, $a_1,a_2,a_3$ are the amplitudes of the three-state coin registers in the initial state, which satisfy $\left| a_1\right| ^2+\left| a_2\right| ^2+\left| a_3\right| ^2=1$.
2. Apply $U_{msg}$ to the initial state $\left| \Psi \left( t=0\right) \right\rangle$ and generate the final probability distribution for each position, denoted by $P=\left( p_0,p_1,p_2,\cdots p_{N-1}\right)$, where$p_x\left( x\in z_N\right)$ is the probability of the particle in its final state finding at each vertex of the cycle given by Eqs. (7) and  (8).
3. Perform the post-processing algorithm on the obtained probability distribution to generate $N\times s$ hash values. In particular, perform $\left\lfloor p_x\cdot {10}^l\right\rfloor {\rm{mod}}\ 2^s$ on $p_x$. Then concatenate these *s*-bits $h_x\ (x\in z_N)$ together to form the final hash value${h=h}_0\Vert h_1\Vert h_2\Vert \cdots \Vert h_{N-1}$ .

## Security analysis

In this section, we will discuss the security of the proposed scheme against the preimage attack, the force search attack and the force search attack.

### Preimage resistance

Hash functions should have one-wayness, which guarantees the security of plaintext information from malicious adversaries, i.e., the adversary cannot guess the original message through the hash value with a non-negligible probability. We define a message space ${\mathbb {M}}$, quantum state space ${\mathbb {Q}}$ and hash space ${\mathbb {H}}$. Function mapping $f:{\mathbb {M}}\rightarrow {\mathbb {Q}}\rightarrow {\mathbb {H}}$ firstly maps the message ${msg}\in \{0,1\}^L$ to the quantum state of a quantum system, denoted by $\left| \Psi \right\rangle _{final}$, then to the hash space $h\in \{0,1\}^m,m<L$, which is a classical-classical mapping. Moreover, the inversion of this mapping is tough to accomplish with two shields. The first shield is using the post-processing algorithms to obtain the hash value by truncation arithmetic and modular arithmetic on the probability of vertexes, which obviously is a many-to-one mapping. The second shield is based on the irreversibility of quantum measurement on the final state, which breaks the linear relationship between the final state and the initial state, even if the adversary takes the hash value in hand and has a priori knowledge of initial state. For example, assume that the message length *L* is disclosed to the adversary, there is no efficient polynomial time algorithm *A*(*f*(*x*)) to try all of $2^L$ guesses, which satisfies Eq. (1). Therefore, when the message space is sufficiently large, the adversary cannot backtrack the original message by hash value with a non-ignorable probability.

### Force search attack resistance

Cryptographic hash functions are designed to keep their output as short as possible, but it is still be difficult to find collisions. There is a powerful attack for hash functions acting on hash value, $birthday\ attack$^31^. The birthday attack can find the collision of the target hash *h* with a $50\%$ probability of only having $O(\sqrt{sN})$ expected times. For classical computers, the general hash length of $\frac{sN}{2}\ge 128$ can satisfy the condition of cryptographic security. Because of the extensibility of the hash length in our proposed scheme, we can easily construct hash functions that satisfy the security conditions.

### Forgery attack resistance

Here forgery attack means that a valid message-hash pair can be obtained from other message-hash pairs that are of equal or variant lengths. The forgery attack is closely related to the second-preimage resistance of the hash function. To demonstrate such attack on the original scheme, following procedure is introduced.

Periodic quantum walk on a cycle is defined above in definition *Periodic  quantum   walk  on   a   cycle*. If there is more than one quantum walk with a finite period, it will be easy to generate collisions in all DQW-based hash functions. Specifically, assume a quantum walk algorithm with a chosen evolution operator $U_1$ (executed when the message bit is 1) has a finite period *T*. In that case, ${U_1}^T\left| \Psi \left( t=0\right) \right\rangle =I\left| \Psi \left( t=0\right) \right\rangle \ =\left| \Psi \left( t=0\right) \right\rangle$, so for a message $msg=10 \ldots 10$, any $nT,n\in \{1,2,3\ldots \}$ message bits can be inserted into the message string, then a collision can be formed as

9 $$\begin{aligned} U_{msg}=U_1U_0\cdots U_1U_0=U_1U_0\cdots \underset{I}{{\underbrace{U_1{\cdots U}_1}}}{\cdots U}_1U_0. \end{aligned}$$

Anyone can easily construct a collision at this point, making such algorithms no longer satisfy the collision resistance property. Therefore, when designing a hash function based on quantum walks, proper parameters should be selected to avoid this situation. The coin operator of controlled alternate lively quantum walk on a cycle is selected from Grover type of matrix set in A, which will provide strict security and collision resistance. Consequently, collisions may only be affected by computational precision or by truncation and modular operations.

## Statistical performance analysis

It is difficult to evaluate the security of the proposed scheme by rigorous mathematical proof. So widely accepted statistical tests can be used to evaluate the performance and feasibility of the scheme. We take the same public arxiv Dataset as Ref.^15^ used and randomly select from about 2 million records as the input string. Note that since DQW-based hash functions are classical input and classical output, the hash value of a message in classical computation is usually generated and stored in ASCII format.

In “Force search attack resistance”, the security lower bound for the birthday attack of the hash function is given. To ensure the security of the scheme, we generate $Len = sN>256$-bit hash values, i.e., $Len\in \{296,264\}$. As with other existing DQW-based hash functions, two instances of QHFL share the same *l* value, and the remaining parameters are $C=Grov$, $\alpha _1=\alpha _2=\alpha _3=\frac{1}{\sqrt{3}}$. The statistical performance analyses include: Collision resistance, sensitivity, diffusion and confusion propertiy, uniform distribution. The test results will be compared mainly with the typical scheme QHFM^15^.

### Collision resistance

Collision resistance is an important property of cryptographic hash functions. Such property of hash functions can be quantified by collision test. In general, collision resistance is assessed by two types of tests, $T_1$ and $T_2$. In the test $T_1$, a random input message is selected and computed to obtain an ASCII hash value $h_1$. In the test $T_2$, a random bit reversal is performed on the message in the test $T_1$, and the corresponding hash value $h_2$ is also generated and stored in ASCII format by $h_1=\{b_1,b_1\ldots ,b_s\}$ and $h_2=\{{b'}_1$,${b'}_1\ldots ,{b'}_s\}$, respectively, where $b_i$ and ${b'}_i$ are the *i*th ASCII characters, and $s=\frac{Len}{8}$ represents the size of the hash value in ASCII format.

Moreover, we compare the number of characters of $h_1$ and $h_2$ with the same value at the same location, i.e., the number of hits, and calculate as follows:

10 $$\begin{aligned} \omega =\sum _{i}^{N}{\delta (T(b_i),T({b'}_i))}, \end{aligned}$$

where $$\delta (x,y)=\left\{ \begin{matrix} 1,&{} x=y\\ 0,&{} x \ne y \end{matrix}\right.$$ indicates that $b_i$ and ${b'}_i$ are converted to the corresponding decimal values with $T(b_i)$, $T({b^\prime }_i)\in \{0,1,2,\ldots ,255\}$.

Through $J=10000$ times independent experiment of drawing records from the arxiv dataset and assuming that the hash value distribution is uniform and random, the theoretical number of tests can be given by the binomial distribution formula:

11 $$\begin{aligned} W_t\left( \omega \right) =J\times {Prob}^t\{\omega \}=J\frac{s!}{\omega !\left( s-\omega \right) !}\left( \frac{1}{2^8}\right) ^\omega {(1-\frac{1}{2^8})}^{s-\omega }, \end{aligned}$$

where $\omega =0,1,\ldots ,s$, ${Prob}^t\{\omega \}$ is the probability distribution of the theoretical number of hits (the probability distribution of the experimental number of hits is denoted by ${Prob}^e\{\omega \})$), and the distance with the theoretical value can be measured by means of $Kullback-Leibler$ relative entropy (KL divergence)^32^

12 $$\begin{aligned} D({Prob}^t\{\omega \}\Vert {Prob}^e\{\omega \})=\sum _{\omega =0}^{s}{\frac{W_t\left( \omega \right) }{J}\log _2{\left( \frac{W_e\left( \omega \right) }{J\times {Prob}^t\{\omega \}}\right) }}. \end{aligned}$$

The smaller $D({Prob}^t\{\omega \}\Vert {Prob}^e\{\omega \})$ is, the closer $W_t\left( \omega \right)$ and $W_e\left( \omega \right)$ are, which means that the hash function has the exceptional collision resistance property.

The collision resistance property can also be quantified by the absolute difference between two hash values (i.e., the distance between two hash values):

13 $$\begin{aligned} d=\sum _{i}^{s}{\vert T(b_i)-T({b'}_i)\vert }. \end{aligned}$$

The experimental mean difference per byte of two hash values can be calculated by *d*, then obtain the absolute difference between it and its theoretical value is denoted by $\triangle {\bar{d}}=\vert {{\bar{d}}}^t-{{\bar{d}}}^e\vert$. Since the spatial distribution of ASCII characters is independent and uniform, and ${{\bar{d}}}^t$ can be calculated by the following equation^33^:

14 $$\begin{aligned} {{\bar{d}}}^t=E\left( T\left( b_i\right) -T\left( {b^\prime }_i\right) \right) =\frac{1}{3}\times Lev\approx 85.3, \end{aligned}$$

$Lev=256$ is the number of levels. As the number of experiments increases, ${{\bar{d}}}^e$ will converge to ${{\bar{d}}}^t$, which means better property of collision resistance. As we can see from Table 1, two instances of our scheme have a smaller $D({Prob}^t\{\omega \}\vert \vert {Prob}^e\{\omega \})$ and $\Delta {\bar{d}}$ compared with those in QHFM, which implies that our scheme has better performance in the collision test.

So far this section has focused on collision test to assess the collision resistance. The main criterion for collision resistance of a hash function is that the hash value has less number of hits when a random bit reversal is performed on the input message. When the discrete-time quantum walks perform on an even-length cycle starting in the position $\left| 0\right\rangle$ after odd steps, then the probability at the even vertexes is zero, while the odd vertex is not zero. This periodic behavior is similar on lively quantum walk with an odd lively parameter on an even-length cycle $\tau$. The issue that emerges from these pattern is that the collision test can not pass. However, the lively quantum walks with even lively parameters $\tau$ do not show such periodic pattern for both even-length cycles and odd-length cycles. It can thus be suggested that the quantum hash function proposed in our paper can satisfy multi-level security, i.e., it can output variable-length hash values. By contrast, most quantum hash functions based on discrete-time quantum walks cannot satisfy this property, e.g., they cannot output a 256-bit hash value^14–20^.

**Table 1:** Collision resistance test results.

Instance		Numbers of hits $\omega$					${D}({{Prob}}^{t}\{{\omega }\}\Vert {{Prob}}^{e}\{{\omega }\})$	$\triangle \bar{{d}}$
		0	1	2	3	4		
QHFL-296	$W_e\left( \omega \right)$	8637	1278	81	4	0	0.00009977	0.15
	$W_t\left( \omega \right)$	8652	1255	89	4	0		
QHFM-296^15^	$W_e\left( \omega \right)$	8662	1250	85	2	1	0.00001848	0.13
	$W_t\left( \omega \right)$	8652	1255	89	4	0		
QHFL-264	$W_e\left( \omega \right)$	8784	1133	82	1	0	0.00024273	0.02
	$W_t\left( \omega \right)$	8788	1137	71	3	0		
QHFM-264^15^	$W_e\left( \omega \right)$	8853	1074	73	0	0	0.00072578	0.11
	$W_t\left( \omega \right)$	8788	1137	71	3	0		

### Sensitivity

An effective hash function needs to be extremely sensitive to the initial input, which means that any slight change in the input message will produce a hash value completely unrelated to the result of the original message. To verify the sensitivity of the proposed scheme, four experiments were designed and calculated with the following input information:

- *M*1: Randomly draw a record from the dataset
- *M*2: Randomly flip the bits of message *M*1 at a location
- *M*3: Randomly insert a random bit at a location in message *M*1
- *M*4: Delete a bit at a random location in message *M*1

The hash values corresponding to the above four input messages are $H_{Mj},j=1,2,3,4$. Taking the instance that the output hash value length is $L = 296$-bit, the hexadecimal format of the four hash values is described as follows:

$$H_{M1}$$:D8CCADC2F92994BCBA291E6E790B848ED2ECF9A4D406D00DB40

084B7129C9CADB8BE6A504D

$$H_{M2}$$:AD8E79F3A902AC2AA9C16FD35389ECA58C5ACAE9243EC67CB5F

1FF68576C5C2A5252F54B47

$$H_{M3}$$:712DBD57C4300F84478C36FF9FAC6CF46DC2463EEA006FEA237

F1679FC93E019AA42FC017D

$$H_{M4}$$:C8555E380EA9854F3F913CA2D30F86F1AA386748E0345E6F223

9FC84D1C986511F00F23AB0

**Figure 1:**
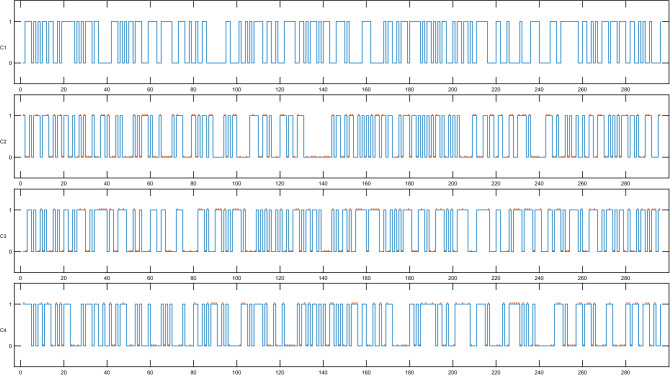
Hash values in the binary format generated by QHFL-296. The symbol * represents a different bit between ${H}_{{M}{2}}$ and ${H}_{{M}{2}}-{H}_{{M}{4}}$.

The binary format of the hash values corresponding to the input messages *M*1–*M*4 is shown in Fig. 1, which demonstrates that even a slight change of the input message can also cause the new hash value to have a large deviation from the original hash value. This result further demonstrates that QHFL can satisfy the one-wayness well.

Since the hash value of the proposed scheme is calculated by the probability distribution of each vertex in the final state on the cycle, the distance between the discrete probability distribution corresponding to the modified message and the original message, respectively, can also be used as an indicator to measure the sensitivity of DQW-based hash functions.

For two discrete probability distributions $P=(p_1,p_2,\ldots p_N)$ and $Q=(q_1,q_2,\ldots q_N)$ on a finite alphabet of size *N*, the distance between them can be measured by $Jensen-Shannon\ divergence$ (JSD)^34^, which is denoted by JSD in the sensitivity test. Different from KL divergence, it is a symmetric function, defined everywhere, with values between 0 and 1. The larger JSD value indicates the farther distance between the two probability distributions. KL divergence can be modified slightly to obtain the JSD formula

15 $$\begin{aligned} JSD({Prob}^{M1}\parallel {Prob}^{Mk})=\frac{1}{2}D({Prob}^{M1}\parallel {Prob}^{Mk})+\frac{1}{2}D({Prob}^{Mk}\parallel {Prob}^{M1}), \end{aligned}$$

where *Mk* denotes a modified message with a range of $k\in \{2,3,4\}$, and $D(\cdot \parallel \cdot )$ denotes the KL divergence in Equation 12. After repeating 2048 independent sensitivity test experiments, as listed in Table 2, the QHFL scheme is more sensitive to messages in comparison with the QHFM scheme.

**Table 2:** JSD-sensitivity test results.

Instance	*M*2	*M*3	*M*4
QHFL-296	0.0810050584976550	0.136948519786094	0.137304527477550
QHFM-296	0.0433801323884277	0.0578510007648174	0.0574491187555278

### Diffusion and confusion property

Diffusion and confusion methods, first introduced by Shannon^35^, can effectively impede the statistical analysis of encryption algorithms. In the context of a hash function, diffusion (often referred to as the avalanche effect) allows any slight impact on the original message and is propagated throughout the whole output, which means that the output hash is highly correlated with the input text. For the binary format of the input and output hash values, any change in the original message bit leads to a change in each bit of the hash value with a $50\%$ probability. Confusion attempts to hide the input message and the relation between the key and the output hash value, which means that it is difficult for an adversary to break the security of the hash function by means of statistical analysis.

To evaluate the diffusion and confusion performance of QHFL, we independently repeat the following experiments $J=10,000$ times. In the *i*th experiment, the original message *M*1 generates its corresponding hash value *h*, and then randomly selects and flips a bit in *M*1 to obtain a new hash value $h'$. The new *L*-bit hash value is then compared with the original message, and followed by calculating their Hamming distance $B_i=\sum _{k=1}^{\vert h\vert }{h(k)\bigoplus h'(k)}$ between the two hash values per experiment. Four statistical indicators can be further calculated to evaluate the diffusion and confusion property of the proposed hash function:

- Mean number of bits changed ${\bar{B}}=\frac{\sum _{i=1}^{J}B_i}{J}$ bit
- Mean probability of change per bit $P=\ \frac{{\bar{B}}}{\vert h\vert }\times 100\%$
- Standard deviation of the changed bit number $\Delta B=\sqrt{\frac{1}{J-1}\sum _{i=1}^{J}{(B_i-B)}^2}$ bit
- Standard deviation of the mean change per bit probability $\Delta P=\sqrt{\frac{1}{J-1}\sum _{i=1}^{J}{(P_i-P)}^2}\times 100\%$

The ideal values of ${\bar{B}}$ and $P(\%)$ are $\frac{L}{2}$ and $50\%$, respectively. Meanwhile, a smaller standard deviation of $\Delta B$ and $\Delta P(\%)$ can also explain the stability of the diffusion and confusion effects. Comparison between QHFL and QHFM is listed in Table 3. It can be concluded from the results that the $\Delta B$ and $\Delta P(\%)$ of QHFL are slightly less than or approximately equal to QHFM. In addition, it is obvious that ${\bar{B}}$ and $P(\%)$ of QHFL are approximate to the ideal values, which are on the same level as QHFL.

Differential analysis exploits weaknesses in the diffusion and confusion property and is based on the analysis of the correlation between some input differences and the output differences. These differential attacks can pose a serious threat to the security of hash functions as well. If there is a strong correlation between different inputs and outputs, one can easily detect message collisions. Therefore, diffusion and confusion is a strong indicator for assessing the resistance of hash functions against the differential analysis attacks. Excellent diffusion and confusion property evaluated from tests indicate that the proposed scheme has robust resistance and reliability to differential analysis attack.

**Table 3:** Results of diffusion and confusion tests.

Instance	${\bar{B}}$	$P(\%)$	$\Delta B$	$\Delta P(\%)$
QHFL-296	148.19	50.06	8.55	2.89
QHFM-296	147.91	49.97	8.81	2.98
QHFL-264	132.03	50.01	8.11	3.07
QHFM-264	131.96	49.98	8.16	3.09

### Uniform distribution

**Figure 2:**
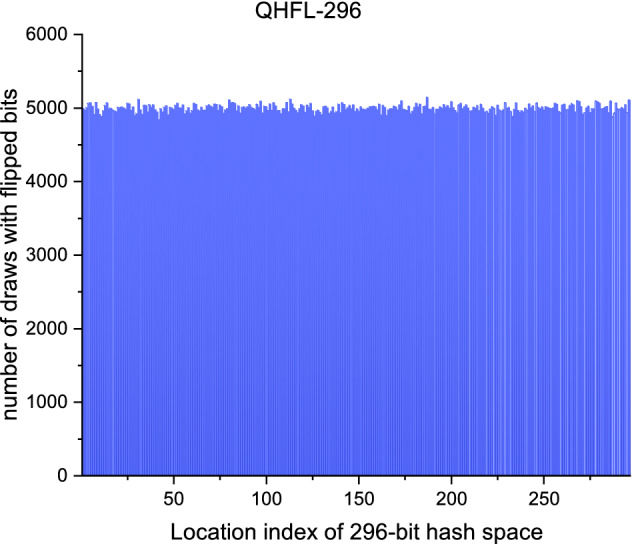
Histogram of the 296-bit hash space.

In general, the uniform distribution of output hash values over the compression range is one of the most critical security features. When the output space of a hash function is uniformly distributed, the input message will hide the statistics of the original message after diffusion and confusion. As shown in Fig. 2, we count the number of tests to flip a hash bit at each location ,and the result is visually described in the form of a histogram, which proves that the QHFL scheme is able to resist statistical analysis attacks.

## Computing complexity

For the case where QHFL with *L* bits input and *O*(*N*) bits output, it is easy to perform both in the quantum and classical computer, specifically, only up to *O*(*LN*) time complexity is required in the classical case, and at most, $O (\lceil \log _2{N}\rceil )$-sized elementary gates along with memory are necessary for the quantum case. In the classical context, it is trivial to obtain the time and space complexity of these schemes by analyzing the number of arithmetic operations performed during the entire hash process^15^. Such schemes can also be efficiently implemented under quantum computing models by performing controlled alternate lively quantum walks, such that we can give the design of quantum circuit for QHFL by some trivial alterations on the quantum circuit of DQW.

### Classical time and space complexity

The quantum evolution at each step of controlled alternate lively quantum walk on an *N*-length cycle is simulated by matrix multiplication. The quantum state at the *t*-th step can be denoted by $\left| \Psi (t)\right\rangle =\sum _{x\in z_N}{a_{x,t}^c\left| x,c\right\rangle }$ or in Eq. (5). Each step performs a quantum evolution via a unitary operator $U_1=S_1\left( I_N\otimes C\right)$ or $U_0=S_0\left( I_N\otimes C\right)$ controlled by message bits, where $S_1$ and $S_0$ represent the shift operators of different $\tau$, *C* is the coin operator, denoted by $$\left[ \begin{matrix}C_1\\ C_2\\ C_3\\ \end{matrix}\right]$$. Where $C_i=\sum _{k=1}^{3}{C_{i,k}\left| i\right\rangle \left\langle k\right| }$,$i\in \{1,2,3\}$, ${C_{i,k}}$ are the elements in the *i* th row and *k* th column of the matrix *C*. After combining Eqs. (5) and (6), the transformation of coin operator acting on $\left| \psi _{x,t}\right\rangle$ can be formulated as

16 $$\begin{aligned} \begin{aligned} \left| \Psi \left( t+1\right) \right\rangle&=\sum _{x\in z_N} C_1\left[ \begin{matrix}\left( a_{x+1,t}^1\right) ^\dagger \\ \left( a_{x+1,t}^2\right) ^\dagger \\ \left( a_{x+1,t}^3\right) ^\dagger \\ \end{matrix}\right] \left| x+1,0\right\rangle +\sum _{x\in z_N} C_2\left[ \begin{matrix}\left( a_{x-1,t}^1\right) ^\dagger \\ \left( a_{x-1,t}^2\right) ^\dagger \\ \left( a_{x-1,t}^3\right) ^\dagger \\ \end{matrix}\right] \left| x-1,1\right\rangle \\&\quad +\sum _{x\in z_N} C_3\left[ \begin{matrix}\left( a_{x+\tau ,t}^1\right) ^\dagger \\ \left( a_{x+\tau ,t}^2\right) ^\dagger \\ \left( a_{x+\tau ,t}^3\right) ^\dagger \\ \end{matrix}\right] \left| x+\tau ,2\right\rangle , \end{aligned} \end{aligned}$$

where $x+1$, $x-1$, and $x+\tau$ are all calculated by modular arithmetic under modulus *N*. According to Eq. (16), the amplitude of the quantum state $\left| \Psi \left( t\right) \right\rangle$ is given by

17 $$\begin{aligned}&\left[ \begin{matrix}a_{x+1,t}^1&{}a_{x+1,t}^2&{}a_{x+1,t}^3\\ \end{matrix}\right] ^\dagger =\left[ \begin{matrix}a_{x+1,t}^1&{}a_{x+1,t}^2&{}a_{x+1,t}^3\\ \end{matrix}\right] ^\dagger +{\left[ \begin{matrix}a_{x,t-1}^1&{}a_{x,t-1}^2&{}a_{x,t-1}^3\\ \end{matrix}\right] ^\dagger C}_1\\&\left[ \begin{matrix}a_{x-1,t}^1&{}a_{x-1,t}^2&{}a_{x-1,t}^3\\ \end{matrix}\right] ^\dagger =\left[ \begin{matrix}a_{x-1,t}^1&{}a_{x-1,t}^2&{}a_{x-1,t}^3\\ \end{matrix}\right] ^\dagger +{\left[ \begin{matrix}a_{x,t-1}^1&{}a_{x,t-1}^2&{}a_{x,t-1}^3\\ \end{matrix}\right] ^\dagger C}_2\\&\left[ \begin{matrix}a_{x+\tau ,t}^1&{}a_{x+\tau ,t}^2&{}a_{x+\tau ,t}^3\\ \end{matrix}\right] ^\dagger =\left[ \begin{matrix}a_{x+\tau ,t}^1&{}a_{x+\tau ,t}^2&{}a_{x+\tau ,t}^3\\ \end{matrix}\right] ^\dagger +{\left[ \begin{matrix}a_{x,t-1}^1&{}a_{x,t-1}^2&{}a_{x,t-1}^3\\ \end{matrix}\right] ^\dagger C}_2. \end{aligned}$$

The calculation process of the QHFL scheme consists of two parts of arithmetic operations. The first part is to update the quantum state amplitudes, and the second is to use multiplication and modular arithmetic to obtain the final hash value. Since the liveliness parameter $\tau$ is controlled by each message bit, a QHFL with *L* steps has the same complexity as the controlled alternate lively quantum walk on an *N*-length cycle with message length *L*. Given the quantum state amplitude at the $t-1$th step and the position *x*, the new quantum state amplitude at the *t*th step can be updated by only 9 multiplications and 9 additions. For a cycle with *N* vertexes, each step will take *O*(*N*) basic arithmetic operations. Obviously, for an input message of *L* bits, the basic operations required for obtaining the value of amplitude only increase by *O*(*LN*). In addition, such computation needs 3*N* registers to store the amplitude values, so the space complexity is *O*(*N*). The time and space complexity of other existing schemes can be analyzed by using similar processes related to the length of the output hash value. As a result, QHFL has the same classical time and space complexity as its peers^13–19,21,23^ except Cao-195^20^.

### Quantum circuit and resource consumption

For the reason of exponential speedups, quantum implementations of the proposed scheme must scale logarithmically with graph scale on which it performs. As a structure with a high degree of symmetry, a small number of parameters can characterize the cycle^36,37^. QHFL, whose underlying model controlled alternate lively quantum walk on the *N*-length cycle can be regarded as the DQW proceeding around a slightly modified *N*-length cycle with additional connections for each transition, such that each vertex on the cycle has three adjacent edges. Then, we give an efficient quantum circuit to implement QHFL by referring to Ref.^36^.

**Figure 3:**
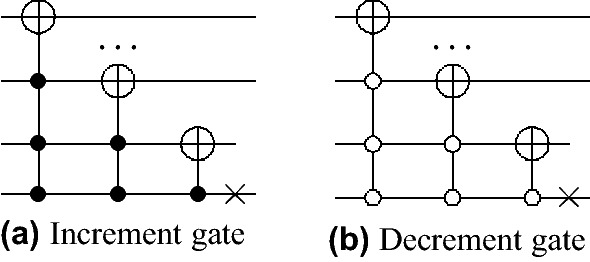
Increment and Decrement gates on *n* qubits.

**Figure 4:**
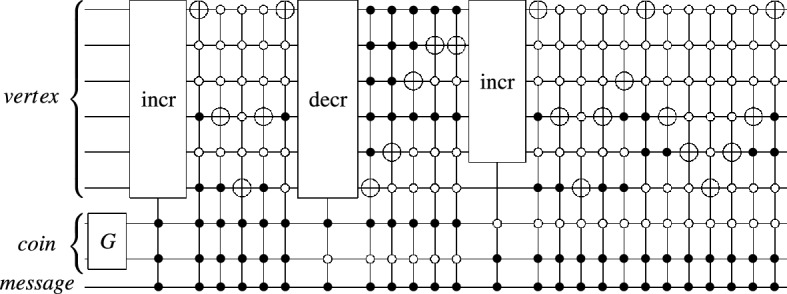
Quantum circuit implementing QHFL with the liveliness parameter $\tau = 2$ alone a 37-length cycle controlled by message bit 1.

**Figure 5:**
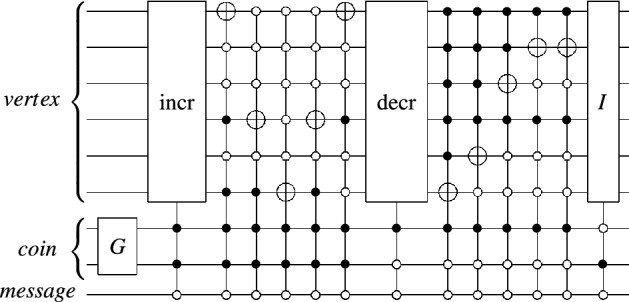
Quantum circuit implementing QHFL with the liveliness parameter $\tau = 0$ alone a 37-length cycle controlled by message bit 0 per step.

There are three associated spaces: the vertex space, in which *x* lies, the coin space, in which *c* lies, the message space, in which *msg* lies. We firstly assign each vertex *x* a bit string in lexicographic order to encode the vertex space, then use $\lceil \log _2{3}\rceil$ (rounded up to a nearest integer) additional qubits to encode the coin space, where the first three bit strings represent the three edges of each vertex. In the end, one qubit is required to encode the message space, with each computational basis $\left| 0\right\rangle$ and $\left| 1\right\rangle$ representing the input message bits 0 and 1, respectively. The shift operator can be implemented by a cyclic permutation gate, which increases or decreases a vertex bit string value into its adjacent vertexes in Fig. 3, and the coin operator is the Grover operator defined on $\lceil \log _2{3}\rceil$ dimensions. Such quantum circuit is made up of generalized CNOT gates, which $O(\lceil \log _2{N}\rceil )$ additional ancillary qubits and $O(\lceil \log _2{N}\rceil )$ elementary gates are also required. An example of the quantum circuit for the implementation of QHFL at per step controlled by per message bit is shown in Figs. 4 and  5.

## Advantage

Through the above analysis, QHFL performs better in tests of statistical performance than other QW-based quantum hash functions. Furthermore, QHFL has some advantages than other CAQW-based quantum hash functions benefit from the structure of controlled alternate lively quantum walks on cycles.

Applying the controlled alternate lively quantum walk as an underlying algorithm for quantum hash functions presents several useful attributes. Firstly, simply changing the liveliness parameters, not just the coin operators of the controlled alternate lively quantum walk employed results in a totally different hash output. Without introducing an additional direction-determine operator as in QHFM, QHFL can implement different quantum hash functions for different purposes by setting unique liveliness parameters, for each purpose. Secondly, the structure of controlled alternate lively quantum walks can be modified, allowing for a longer hash output, simply by changing the cycles on which controlled alternate lively quantum walks perform. As described on the collision resist in previous section, QHFL performed on an even-sized cycle will not result in regular hash values. Consequently, differently from some CAQW-based quantum hash functions^14–20^, it can generate hash values with arbitrary length under suitable conditions.

## Discussion

In this paper, we addressed to designing a new quantum hash function QHFL with variable output length, which is based on the controlled alternate lively quantum walks with variable parameters of liveliness. Its performance and security property are evaluated and compared with the existed state-of-art scheme. The selection criteria of coin operators was also discussed to guarantee the security conditions of QHFL, mainly to avoid collisions. The nature of DQW-based quantum hash functions are that different input messages correspond to completely divergent probability distributions of final quantum state. Thus we adopted an additional indicator JSD to evaluate the sensitivity of the probability distribution to a message. The analyses show that QHFL is with the near-ideal properties of collision resistance, sensitivity, diffusion and confusion property, and hash value uniform distribution property. Moreover, the proposed QHFL is a compatible hash function that can perform efficiently on both a quantum and a classical computer. We also design an efficient quantum circuit implementation of QHFL, which only requires ancillary qubits and elementary quantum gates in logarithmic complexity scaling with cycle size. Remarkably, the proposed scheme has better flexibility than the scheme based on the controlled alternate quantum walks with memory, because it is easy to replace the underlying lively quantum walks by changing the liveliness parameters. Thus, such research can facilitate the construction of novel CAQW-base hash functions without restricting the use of controlled coins. Furthermore, QHFL is suited for various security levels required among different applications.

Research on QW-based quantum hash functions is still in progress. Despite some positive results in the design of quantum hash functions, questions remain. Firstly, all the processes on a classical computer of QHFL are realized by multiplication and addition/subtraction operations, there is abundant room for further progress in efficiency. Secondly, the collision resistance and differential analysis attack resistance of the proposed quantum hash function is analyzed only by some statistical tests. In future work, more efficient constructions of combining other different quantum walks and using a parallel realization are therefore required to explore. And to develop a full analysis of QW-based quantum hash function, additional studies will be needed to perform a rigorous mathematical proof for our scheme.

## Data Availability

The arXiv dataset analyzed in this paper is publicly available at https://www.kaggle.com/Cornell-University/arxiv. The preprocessed dataset and experimental results are available from the corresponding author on reasonable request.

## A Periodicity of lively quantum walks on cycles

The set of Grover-type matrices^30^ can be represented as $M_{Grov}=\{\frac{2}{3}J-P;P\in {\mathscr {P}}\}$ where *J* is a matrix whose elements are all one, ${\mathscr {P}}=\{P_1=I,P_2=\left( 123\right) ,P_3=\left( 132\right) ,P_4=\left( 23\right) ,P_5=\left( 12\right) ,P_6=\left( 13\right) \}$ is the symmetric group $S_3$ corresponding permutation matrices. Therefore, all parametric orthogonal matrices can be regarded as linear combinations of permutation matrices. The single-parameter orthogonal matrix group is described as follows:

18 $$\begin{aligned} \begin{aligned} {\mathscr {X}}_\theta&=\left[ \begin{array}{ccc}\frac{2cos\theta +1}{3}&{}\frac{(1-cos\theta )}{3}+\frac{1}{\sqrt{3}}sin\theta &{}\frac{(1-cos\theta )}{3}-\frac{1}{\sqrt{3}}sin\theta \\ \frac{(1-cos\theta )}{3}-\frac{1}{\sqrt{3}}sin\theta &{}\frac{2cos\theta +1}{3}&{}\frac{(1-cos\theta )}{3}+\frac{1}{\sqrt{3}}sin\theta \\ \frac{(1-cos\theta )}{3}+\frac{1}{\sqrt{3}}sin\theta &{}\frac{(1-cos\theta )}{3}-\frac{1}{\sqrt{3}}sin\theta &{}\frac{2cos\theta +1}{3}\\ \end{array}\right] \\ 0&<\theta <2\pi \end{aligned}, \end{aligned}$$

then we have

1. $${\mathscr {X}}_\theta \cup {\mathscr {Y}}_\theta$$, where
  19 $$\begin{aligned} \begin{aligned} {\mathscr {Y}}_\theta&=\left[ \begin{array}{ccc}\frac{2cos\theta -1}{3}&{}-\frac{(1+cos\theta )}{3}+\frac{1}{\sqrt{3}}sin\theta &{}-\frac{(1+cos\theta )}{3}-\frac{1}{\sqrt{3}}sin\theta \\ \frac{(1+cos\theta )}{3}-\frac{1}{\sqrt{3}}sin\theta &{}\frac{2cos\theta -1}{3}&{}-\frac{(1+cos\theta )}{3}+\frac{1}{\sqrt{3}}sin\theta \\ \frac{(1+cos\theta )}{3}+\frac{1}{\sqrt{3}}sin\theta &{}-\frac{(1+cos\theta )}{3}-\frac{1}{\sqrt{3}}sin\theta &{}\frac{2cos\theta -1}{3}\\ \end{array}\right] \\&0<\theta <2\pi \end{aligned}. \end{aligned}$$
2. $${\mathscr {X}}_\theta \cup {\mathscr {Z}}_\theta$$, where
  20 $$\begin{aligned} \begin{aligned} {\mathscr {Z}}_\theta&=\left[ \begin{array}{ccc}\frac{2cos\theta +1}{3}&{}\frac{(1-cos\theta )}{3}+\frac{1}{\sqrt{3}}sin\theta &{}\frac{(1-cos\theta )}{3}-\frac{1}{\sqrt{3}}sin\theta \\ \frac{(1-cos\theta )}{3}+\frac{1}{\sqrt{3}}sin\theta &{}\frac{(1-cos\theta )}{3}-\frac{1}{\sqrt{3}}sin\theta &{}\frac{2cos\theta +1}{3}\\ \frac{(1-cos\theta )}{3}-\frac{1}{\sqrt{3}}sin\theta &{}\frac{2cos\theta +1}{3}&{}\frac{(1-cos\theta )}{3}+\frac{1}{\sqrt{3}}sin\theta \\ \end{array}\right] \\ 0&<\theta <2\pi \end{aligned}. \end{aligned}$$
3. $${\mathscr {X}}_\theta \cup {\mathscr {W}}_\theta$$,where
  21 $$\begin{aligned} \begin{aligned} {\mathscr {Y}}_\theta&=\left[ \begin{array}{ccc}\frac{2cos\theta -1}{3}&{}-\frac{(1+cos\theta )}{3}+\frac{1}{\sqrt{3}}sin\theta &{}-\frac{(1+cos\theta )}{3}-\frac{1}{\sqrt{3}}sin\theta \\ \frac{(1+cos\theta )}{3}+\frac{1}{\sqrt{3}}sin\theta &{}-\frac{(1+cos\theta )}{3}-\frac{1}{\sqrt{3}}sin\theta &{}\frac{2cos\theta -1}{3}\\ \frac{(1+cos\theta )}{3}-\frac{1}{\sqrt{3}}sin\theta &{}\frac{2cos\theta -1}{3}&{}-\frac{(1+cos\theta )}{3}+\frac{1}{\sqrt{3}}sin\theta \\ \end{array}\right] \\ 0&<\theta <2\pi \end{aligned}. \end{aligned}$$
4. $${{\mathscr {P}}{\mathscr {O}}_\theta ={\mathscr {X}}}_\theta \cup {\mathscr {Y}}_\theta \cup {\mathscr {Z}}_\theta \cup {\mathscr {W}}_\theta$$

Without loss of generality, the necessary condition for the coin operators that satisfy the periodic lively quantum walks when $C\in {\mathscr {X}}_\theta$ is given by

22 $$\begin{aligned} \begin{aligned} \left\{ \begin{matrix} lcm\{c_lp_l:0<l\le N-1\},\frac{2l}{n}=\frac{m_l}{p_l},gcd{\left( m_l,p_l\right) }=1,&{}\\ c_l=1,if\ m_l\ is\ even\ and\ c_l=2,if\ m_l\ \ is\ odd,\ if\ C=I \\ 3,if\ C\in \{P_2,P_3\}\\ lcm\{3,q\}, if\ N=3\ and\ \theta =\frac{2m\pi }{q},\frac{m}{q}\in {Q},gcd{\left( 2m,q\right) }=1,C\notin \{I,P_2,P_3\}\\ lcm\{3,2q\}, if\ N=3\ and\ \theta =\frac{(2m+1)\pi }{q},\frac{(2m+1)}{q}\in {Q},gcd{\left( 2m+1,q\right) }=1,C\notin \{I,P_2,P_3\}\\ \infty , otherwise \end{matrix}\right. \end{aligned} \end{aligned}$$

Where $lcm\{\cdot ,\cdot \}$ represents the least common multiple, and $gcd{\left( \cdot ,\cdot \right) }$ represents the greatest common divisor. For example, we can consider two special coin operators in ${\mathscr {X}}_\theta$ with parameters $\theta =\frac{\pi }{2}$ and $\theta =\frac{3\pi }{2}$, whose corresponding coin operators are described as follows: $$\left[ \begin{array}{ccc}\frac{1}{3}&{}\frac{1+\sqrt{3}}{3}&{}\frac{1-\sqrt{3}}{3}\\ \frac{1-\sqrt{3}}{3}&{}\frac{1}{3}&{}\frac{1+\sqrt{3}}{3}\\ \frac{1+\sqrt{3}}{3}&{}\frac{1-\sqrt{3}}{3}&{}\frac{1}{3}\\ \end{array}\right]$$, and $$\left[ \begin{array}{ccc}\frac{1}{3}&{}\frac{1-\sqrt{3}}{3}&{}\frac{1+\sqrt{3}}{3}\\ \frac{1+\sqrt{3}}{3}&{}\frac{1}{3}&{}\frac{1-\sqrt{3}}{3}\\ \frac{1-\sqrt{3}}{3}&{}\frac{1+\sqrt{3}}{3}&{}\frac{1}{3}\\ \end{array}\right]$$. Their corresponding periods are given by $$\left\{ \begin{matrix} 12,&{}N=3 \\ \infty ,&{}N \ne 3 \end{matrix}\right.$$.
